# Complete Genome Sequences of Field Isolates of *Mycobacterium bovis* and *Mycobacterium caprae*

**DOI:** 10.1128/genomeA.00247-15

**Published:** 2015-06-25

**Authors:** José de la Fuente, Iratxe Díez-Delgado, Marinela Contreras, Joaquín Vicente, Alejandro Cabezas-Cruz, Marina Manrique, Raquel Tobes, Vladimir López, Beatriz Romero, Lucas Domínguez, Joseba M. Garrido, Ramón Juste, Christian Gortazar

**Affiliations:** a SaBio, Instituto de Investigación en Recursos Cinegéticos IREC (CSIC-UCLM-JCCM), Ciudad Real, Spain; b Department of Veterinary Pathobiology, Center for Veterinary Health Sciences, Oklahoma State University, Stillwater, Oklahoma, USA; c Centro de Vigilancia Sanitaria Veterinaria (VISAVET), Facultad de Veterinaria, Universidad Complutense de Madrid, Madrid, Spain; d Center for Infection and Immunity of Lille (CIIL), INSERM U1019, CNRS UMR 8204, Université de Lille, Institut Pasteur de Lille, Lille, France; e Oh no sequences! Research Group, Era7 Bioinformatics, Granada, Spain; f Departamento de Sanidad Animal, Facultad de Veterinaria, Universidad Complutense de Madrid, Madrid, Spain; g Instituto Vasco de Investigación y Desarrollo Agrario (NEIKER), Departamento de Sanidad Animal, Vizcaya, Spain

## Abstract

Here we report the complete genome sequences of field isolates of *Mycobacterium bovis* and the related mycobacterial species, *Mycobacterium caprae*. The genomes of three *M. bovis* (MB1, MB3, MB4) and one *M. caprae* (MB2) field isolates with different virulence, prevalence, and host distribution phenotypes were sequenced.

## GENOME ANNOUNCEMENT

Animal tuberculosis (TB) is caused by infection with *Mycobacterium bovis* and is closely related to members of the *M. tuberculosis* complex (MTBC) such as *M. caprae* (1, 2). Presently, a large number of *M. tuberculosis*, but few *M. bovis* (except for BCG strains), genome sequences are available (3). Here, we report the complete genome sequences of three field isolates of *M. bovis* and a field isolate of the related mycobacterial species *M. caprae*.

The *M. bovis* spoligotypes SB0339 (MB1, MB4) and SB0134 (MB3) and the *M. caprae* spoligotype SB0157 (MB2) with different virulence, prevalence, and host distribution were obtained from domestic or wild ungulates. The isolates were grown in 15 mL of Middlebrook 7H9 liquid media supplemented with 0.36% sodium pyruvate and 10% OADC (oleic albumin dextrose catalase) for 5 weeks. Chromosomal DNA samples were obtained as previously described (4). Genomic DNA was subjected to mechanical fragmentation using a BioRuptor (Life Technologies, Carlsbad, CA, USA) to obtain DNA fragments of a final average size of about 500 bp. Samples were then used to prepare sequencing-amenable TruSeq libraries (NEB-Next, New England Biolabs, Ipswich, MA, USA). A library was qPCR-quantitated and brought to a final concentration of 10 nM. DNA was then denatured and equilibrated so that a final library concentration of 18 mate pairs was loaded onto a MiSeq Flow Cell version 3 (Illumina, San Diego, CA, USA) and sequenced using a 2 × 250 pair-end sequencing protocol to obtain more than 400-fold high-quality coverage (1.9 to 2.5 Gb) with 84% of the bases showing a Q30 factor >30. Reads were finally split according to bar codes and used for analysis. High-quality overlapping reads of each pair were merged using FLASH (5) and then assembled using Velvet (6) with a *k* value of 97. Assembled contigs were annotated using BG7 (7). For annotation, a set of 191,017 reference proteins was used, including (a) all Uniprot proteins from *M. bovis* and *M. tuberculosis*, (b) a set of bacterial antibiotic resistance-related Uniprot proteins selected using the GO annotation terms “antibiotic resistance” and “response to antibiotic” and a selection of proteins based on similarity to the proteins of ARDB (8), and (c) all Uniprot proteins with an enzyme code (EC) from MTBC.

The sequenced genomes consisted of 4,275,214 (MB1), 4,288,871 (MB2), 4,259,788 (MB3), and 4,255,612 (MB4) bp. All genomes showed a high GC content: 64.82% (MB1), 65.36% (MB2), 65.10% (MB3), and 65.54% (MB4). The characteristic global genome stability of *M. tuberculosis* was also present in these four genomes, while variability appeared to be concentrated in membrane proteins with repetitive sequence motifs, which bear frequent mutations that are probably related to selective pressures imposed by the host immune system.

The availability of genomes from field isolates of *M. bovis* and *M. caprae* will allow comparative analysis to other mycobacteria species to expand the study of the evolution and host specificity of *Mycobacterium* spp. and to find correlates with phenotypic variation with implications for TB disease risk assessment and control.

### Nucleotide sequence accession numbers.

The *M. bovis* MB1, MB3, and MB4 genome sequences were deposited in the European Nucleotide Archive under the accession numbers CDHF01000001 to CDHF01000049, CDHH01000001 to CDHH01000094, and CDHE01000001 to CDHE01000118, respectively. The *M. caprae* MB2 genome sequence was deposited in the European Nucleotide Archive under the accession numbers CDHG01000001 to CDHG01000059.
